# Effect of corticosteroid injections versus physiotherapy on pain, shoulder range of motion and shoulder function in patients with subacromial impingement syndrome: A systematic review and meta-analysis

**DOI:** 10.4102/sajp.v72i1.318

**Published:** 2016-09-27

**Authors:** Marlette Burger, Carly Africa, Kara Droomer, Alexa Norman, Chloé Pheiffe, Anrich Gericke, Adeeb Samsodien, Natasha Miszewski

**Affiliations:** 1 Physiotherapy Division, Faculty of Medicine and Health Sciences, Stellenbosch University, South Africa

## Abstract

**Background:**

Subacromial impingement syndrome (SIS) is one of the most common causes of shoulder pain. Limited research has been conducted into the efficacy of corticosteroid injections (CSIs) compared to physiotherapy in the management of SIS.

**Objective:**

To critically appraise and establish the best available evidence for the effectiveness of CSI in comparison with physiotherapy for the management of pain, shoulder range of motion (ROM) and shoulder function in patients with SIS.

**Methodology:**

Seven databases were searched from inception to February 2016, namely PubMed, Science Direct, EBSCO Host: SPORTDiscus, EBSCO Host: CINAHL, Cochrane, Scopus and PEDro. The main search terms were shoulder impingement syndrome and/or subacromial impingement syndrome, corticosteroid injections and/or steroid injections, physical therapy and/or physiotherapy. Only randomised controlled trials (RCTs) were considered for inclusion. The articles were appraised according to the PEDro scale. The Revman^©^ Review Manager Software was used to combine the results of shoulder function and the data were illustrated in forest plots.

**Results:**

The PEDro scores of the three RCTs that qualified for this review ranged from 7 to 8/10. There is Level II evidence suggesting that besides a significant improvement in shoulder function in favour of CSI at 6–7 weeks follow-up (*p* < 0.0001), no evidence was found for the superiority of CSIs compared with physiotherapy for pain, ROM and shoulder function in the short- (1–3 months), mid- (6 months) and long term (12 months).

**Conclusion:**

In patients with SIS only a short term significant improvement in shoulder function was found in favour of CSIs.

## Introduction

Subacromial impingement syndrome (SIS) is one of the most common causes of shoulder pain and is defined as a narrowing of the subacromial space with subsequent impingement of the bursa, long head of the biceps tendon, the rotator cuff tendons and the coracoacromial ligament (Umer, Qadir & Azam 2012). SIS can present in more than one form and although impingement syndrome represents the supraspinatus muscle impinging beneath the acromion, it does not fully describe the extent of the underlying shoulder pathology (Cummins, Sasso & Nicholson 2009). SIS may range from subacromial bursitis and/or inflammation in the rotator cuff tendons to degeneration of the bursa or rotator cuff tendons to full-thickness rotator cuff tendon tears or more serious degenerative joint disease within the shoulder girdle (Harrison & Flatow 2011; Michener, McClure & Karduna 2003). The consequences of SIS include moderate to severe shoulder pain that is worsened by movement. This condition often results in functional limitation in flexion and abduction range and may cause loss of function and disability (Akgün, Birtane & Akarırmak 2004; Crawshaw *et al*. 2010; Michener *et al*. 2003).

There is a wide variety of conservative treatments for SIS ranging from different physiotherapy modalities such as joint mobilisation techniques and strengthening exercises, adaptations of daily activities, non-steroidal anti-inflammatory drugs (NSAIDs) as well as steroid injections (Dorrestijn *et al*. 2009). Subacromial corticosteroid injection (CSI) is a popular SIS treatment method amongst orthopaedists, rheumatologists and general practitioners (Rhon, Boyles & Cleland 2014). This method is regarded as an inexpensive and effective way to both diagnose and treat symptomatic rotator cuff disease and SIS (Gruson, Ruchelsman & Zuckerman 2008). Therapeutic effects of CSI on pain, inflammation and range of motion (ROM) have mostly been observed as being limited to a short-term effect (Akgün *et al*. 2004). However, more recent systematic reviews found limited evidence on the effectiveness of CSIs for SIS compared with placebo injections (Dorrestijn *et al*. 2009; van der Sande *et al*. 2013).

Physiotherapy treatment techniques for SIS focuses on reducing pain, reversing abnormal muscle imbalances and increasing strength, promoting healing as well as increasing pain-free shoulder motion (Michener, Walsworth & Burnet 2004). Physiotherapy techniques used to reduce pain and improve active and passive ROM include joint mobilisation techniques to improve motion at the glenohumeral joint as well as the cervical and upper thoracic spine, pendulum exercises as well as strengthening exercises and soft-tissue mobilisation and stretches (Crawshaw *et al*. 2010; Michener *et al*. 2004; Rhon *et al*. 2014). Reversing abnormal rotator cuff muscle and shoulder stabiliser imbalances plays an important role in the physiotherapy management of SIS. A good balance between the stabilisers and the movers around the shoulder increases patient-reported function and helps to reduce pain (Hanratty *et al*. 2012).

No systematic review has been previously conducted to determine the effect of CSIs compared with physiotherapy in the management of patients with SIS. The purpose of this systematic review was thus to determine the best short-term (1–3 months); medium-term (6 months) and long-term (12 months) approach for the management of SIS by systematically identifying, collating and analysing the current available evidence on the effectiveness of CSIs versus physiotherapy in the treatment of pain, shoulder ROM and shoulder function.

## Methodology

### Search strategy

A total of seven electronic databases, available through Stellenbosch University Library, were searched, namely Pubmed, Science Direct, EBSCO Host: SPORTDiscus, EBSCO Host: CINAHL, Cochrane, Scopus and PEDro. The key search terms used were shoulder impingement syndrome, corticosteroid injections, physical therapy, physiotherapy, steroid injections and subacromial impingement syndrome. Each database received an individual search strategy according to its function. Each database was searched independently by two investigators. Based on the inclusion and exclusion criteria below, investigators independently reviewed the titles, abstracts and full-text articles retrieved in the initial search. The researchers compared the eligible articles selected for inclusion, and disagreements for accepting full-text articles were discussed until consensus was achieved.

### Study inclusion and exclusion criteria

The following inclusion and exclusion criteria were applied:

#### Type of studies

Only randomised controlled trials (RCTs) published in English from inception of the databases until February 2016 were eligible for inclusion in this review.

#### Type of participants

Study participants could include adults (≥18 years), male and/or females, with primary symptoms of moderate or severe unilateral shoulder pain that was made worse with movement and had a non-capsular pattern of restriction. Tests confirming SIS could include a positive Neer or Hawkins–Kennedy impingement test (Petty 2011). RCTs were excluded if they recruited participants who had a history of previous shoulder injuries, for example, previous shoulder dislocations, rotator cuff ruptures or scapula and/or humeral head/neck fractures, adhesive capsulitis, glenohumeral arthritis or previous shoulder surgery.

#### Types of interventions

CSIs including, but not limited to, injections at the midpoint of the acromion as well as in the subacromial space of the symptomatic shoulder.

#### Types of comparisons

Physiotherapy management including, but not confined to, manual stretches, contract–relax techniques, strengthening exercises directed to the shoulder girdle or thoracic or cervical spine, electrotherapy modalities and home advice regarding management and precautions. Physiotherapy management had to include a combination of passive and active joint and soft-tissue mobilisation techniques.

#### Type of outcomes

RCTs had to assess at least one the following three clinical outcomes, namely pain, ROM and shoulder function.

### Evidence hierarchy and methodological appraisal

According to the National Health and Medical Research Council (NHMRC) Hierarchy of Evidence (Merlin, Weston & Tooher 2009), a well conducted RCT, as Level II evidence, is appropriate for the purpose of answering an intervention question in a systematic review. The PEDro scale was used to determine the methodological quality and potential sources of bias of the included studies. The PEDro scale is a valid measure of the methodological quality of clinical trials and is widely used in physiotherapy research (de Morton 2009). Each article was allocated to two researchers who individually appraised the article using the PEDro scale. The researchers compared their results and when a discrepancy occurred, a third researcher was consulted. If agreement was not reached at this point, a group discussion between all eight researchers was held to resolve the matter.

### Data extraction and analysis method

The data were extracted and captured on a Microsoft Excel spreadsheet by one investigator to ensure continuity. The information was cross-checked by the rest of the seven research team members. Mutual consensus amongst the group was ensured after a discussion of the complete data extraction. All data were tabulated into the following categories: citation, type of study, patients (including number of patients and ages), type of intervention, comparisons, outcome measures (including measurement tools, validity and reliability), continuous data (intervention and comparison group), clinical status and implication. The Revman^©^ Review Manager Software, which summarises all the statistics in the form of a meta-analysis (RevMan^©^ Information Management System 2008), was used to combine the results of shoulder function for two of the included articles and the data were illustrated with forest plots. The outcomes for continues data [mean and standard deviation (SD)] were expressed as weighted mean differences (WMD). Heterogeneity amongst the studies was assessed by the *I*^2^ statistic. Studies are regarded as homogeneous if *I*^2^ ≤ 25%, and if *I*^2^ ≥ 75%, the heterogeneity amongst the studies is considered high (Ried 2006). Statistical pooling for pain and ROM was rendered inappropriate owing to heterogeneity amongst reporting of results and were subsequently summarised in a narrative form and illustrated in tables.

### Ethical considerations

We conducted secondary research, thus ethical approval was not required for this review.

## Results

### Search results

**FIGURE 1:**
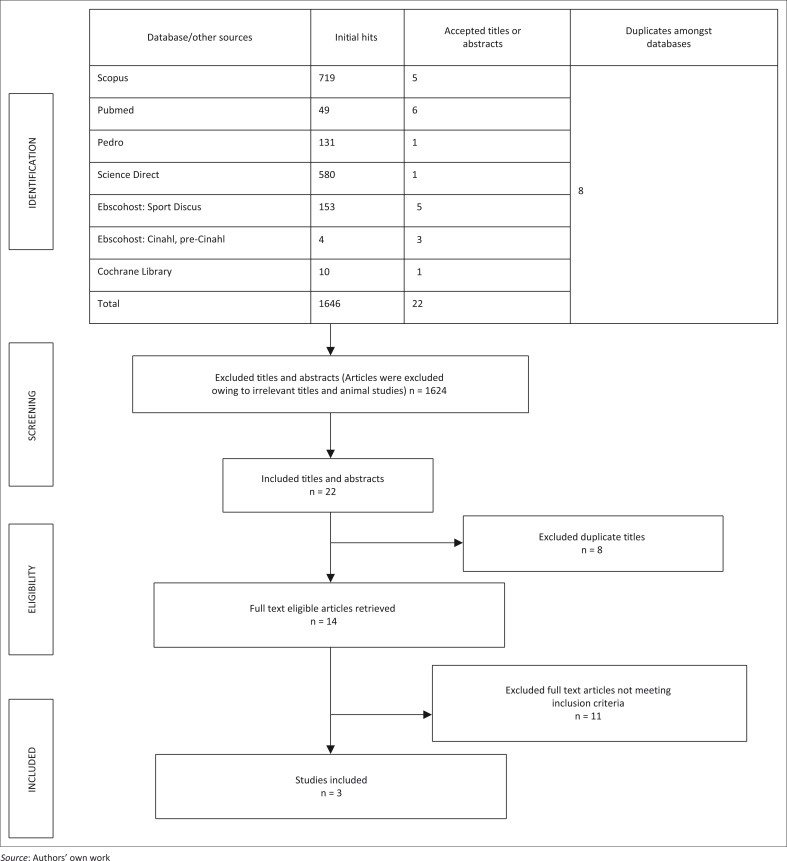
Search results.

The results of the search strategy are presented in a flow chart (see Figure 1). A total of 1646 initial titles were found. Fourteen full-text articles were assessed according to the inclusion and exclusion criteria and of these, three articles (Hay *et al*. 2003; Rhon *et al*. 2014; van der Windt *et al*. 1998) were considered eligible for this systematic review. Reasons for excluding articles were the following: intervention therapy included CSIs as well as physiotherapy (e.g. manual therapy and/or electrotherapy); participants with a history of previous shoulder injuries were included; and comparison therapy did not include a combination of passive and active joint and soft-tissue mobilisation techniques.

### Evidence hierarchy and methodological appraisal

According to the Hierarchy of Evidence set forth by the NHMRC (2005), the three included articles (Hay *et al*. 2003; Rhon *et al*. 2014; van der Windt *et al*. 1998) were classified as Level II. The methodological quality of the three included articles, according to the PEDro scale, ranged between 7/10 and 8/10, with an average score of 7.3/10 (Table 1). The assessors were not blinded in van der Windt *et al*. (1998). Because of the nature of the included articles, it was not possible to blind the therapists or the participants.

**TABLE 1:** Study sample descriptions.

Criteria		Hay *et al*. (2003)	van der Windt *et al*. (1998)	Rhon *et al*. (2014)
Sample size	Corticosteroid injections	104	53	73
	Physiotherapy	103	56	63
Gender	Corticosteroid injections	Male = 42 Female = 44	Male = 47 Female = 25	Male = 38 Female = 35
	Physiotherapy	Male = 51 Female = 53	Male = 59 Female = 33	Male = 29 Female = 34
Age (in years)	Corticosteroid injections	Mean (SD): 57.6 ± 14	Mean (SD): 57.3 ± 10.2	Mean (SD): 42 ± 12
	Physiotherapy	Mean (SD): 57.5 ± 13	Mean (SD): 60.2 ± 10.7	Mean (SD): 40 ± 12
PEDro scores		8/10	7/10	7/10
Country		United Kingdom	The Netherlands	United States
Physiotherapy intervention group	Type of treatment	Advice and instruction on pain relief, active shoulder exercises, home programme, manual therapy and ultrasound.	Passive joint mobilisation, exercise treatment, ice, hot packs, electrotherapy. Did not specify electrotherapy modalities used but stated that they excluded ultrasound.	Joint and soft-tissue mobilisation, manual stretches, contract–relax techniques and reinforcing exercises aimed at the shoulder girdle or thoracic or cervical spine.
	Duration of treatment	20-minute individual physiotherapy sessions for 6 weeks.	30-minute individual sessions for 6 weeks.	Twice a week for 3 weeks.
	Number of sessions	8 sessions	12 sessions	6 sessions
	Other treatment prescribed if clinically indicated during and after the trial	At the end of the trial period, 29 participants of the physiotherapy group received steroid injections, 5 received a further course of physiotherapy and 3 were prescribed analgesics or NSAID’s. Of those participants allocated to the corticosteroid injection group, 11 received another injection, 21 received physiotherapy and 9 were prescribed analgesics or NSAIDs.	Patients were allowed to continue taking medication for pain if they had already started before enrolment. Pain medication was also prescribed during the course of the trial if pain was severe. All other interventions were to be avoided. Did not specify post-trial treatment.	Patients were discouraged to seek additional care during the first month of the study period. Did not specify port-trial treatment.
Corticosteroid injection group	Type of corticosteroid injection	40 mg methylprednisolone mixed with 4% 1 ml lidocaine into the subacromial space. Originally they got one injection and were offered a second one.	Intra-articular injections of 40 mg triamcinolone acetonide was given to patients via the posterior route. 3 Injections were given over a timeframe of 6 weeks.	Intra-articular injections of 40 mg triamcinolone acetonide was given to patients via the posterior route. 3 Injections were given, 1 month apart, over a 1-year timeframe.
	Total number of corticosteroid injections	Patients were given one injection initially and offered a second one if they returned with complaints of pain.	No more than 3 injections were given during the 6 weeks.	A total of 3 injections spaced 1 month apart over the course of 12 months.

*Source*: Authors’ own work

SD, standard deviation.

### Description of study sample and interventions

The summary of the sample descriptions from each study can be found in Table 1. The total sample captured in the three studies was 452. Rhon *et al*. (2014) included slightly younger participants, while the minimum age across the studies was 18 years and the maximum was 65 years. The three studies were conducted in developed countries. Variation was found in the physiotherapeutic interventions across the three studies (Table 1). All three studies included a baseline of exercise therapy and manual therapy. Hay *et al*. (2003) included ultrasound, van der Windt *et al*. (1998) also made use of electrotherapy but specifically excluded ultrasound and added the use of ice and hot packs for pain relief, while Rhon *et al*. (2014) only made use of manual therapy and exercise. The duration of the physiotherapy intervention was well documented in all three studies. The chemical composition of the corticosteroids in van der Windt *et al*. (1998) and Rhon *et al*. (2014) were exactly the same (40 mg triamcinolone acetonide), whereas in Hay *et al*. (2003) 40 mg methylprednisolone mixed with 4% 1 ml lidocaine was used. The site of the CSIs also differed across the three studies. The number of CSIs was specified in van der Windt *et al*. (1998) and Rhon *et al*. (2014) but not in Hay *et al*. (2003).

### Description of outcome measures and assessment times

The outcome measures used in the three articles are summarised in Table 2. Rhon *et al*. (2014) and van der Windt *et al*. (1998) conducted a long-term follow-up (52 weeks) assessment, while Hay *et al*. (2003) conducted the final assessment at 26 weeks.

**TABLE 2:** Outcome measurements used and assessment time intervals.

Outcome measures	Baseline	3–4 weeks	6–7 weeks	13 weeks	26 weeks (6 months)	52 weeks (1 year)
SDQ	van der Windt *et al*. (1998)/Hay *et al*. (2003)	van der Windt *et al*. (1998)	van der Windt *et al*. (1998)/Hay *et al*. (2003)	van der Windt *et al*. (1998)	van der Windt *et al*. (1998)/Hay *et al*. (2003)	van der Windt *et al*. (1998)
VAS	van der Windt *et al*. (1998)/Hay *et al*. (2003)	van der Windt *et al*. (1998)	van der Windt *et al*. (1998)/Hay *et al*. (2003)	van der Windt *et al*. (1998)	van der Windt *et al*. (1998)/Hay *et al*. (2003)	van der Windt *et al*. (1998)
ROM	van der Windt *et al*. (1998)/Hay *et al*. (2003)	van der Windt *et al*. (1998)	van der Windt *et al*. (1998)/Hay *et al*. (2003)	van der Windt *et al*. (1998)	van der Windt *et al*. (1998)/Hay *et al*. (2003)	van der Windt *et al*. (1998)
SPADI	Rhon *et al*. (2014)	Rhon *et al*. (2014)	-	Rhon *et al*. (2014)	Rhon *et al*. (2014)	Rhon *et al*. (2014)
NPRS	Rhon *et al*. (2014)	Rhon *et al*. (2014)	-	Rhon *et al*. (2014)	Rhon *et al*. (2014)	Rhon *et al*. (2014)
GRC	Rhon *et al*. (2014)	Rhon *et al*. (2014)	-	Rhon *et al*. (2014)	Rhon *et al*. (2014)	Rhon *et al*. (2014)

*Source*: Authors’ own work

SDQ, shoulder disability questionnaire; VAS, Visual Analogue Scale; ROM, range of motion; SPADI, Shoulder Pain and Disability Index; NPRS, Numerical Pain Rating Scale; GRC, global rating of change.

### The effect of corticosteroid injections versus physiotherapy

The effect of CSIs versus physiotherapy in the treatment of SIS is shown in Tables 3–7 under the following subheadings: pain, shoulder ROM and shoulder function.

**TABLE 3:** Results for the measurement of pain in van der Windt *et al*. (1998) and Rhon *et al*. (2014).

Variables	van der Windt *et al*. (1998)				Rhon *et al*. (2014)			
	CSI mean (SD) improvement from baseline	Physiotherapy mean (SD) improvement from baseline	Mean (95% CI) difference between groups	*p*	CSI Mean (CI)	Physiotherapy Mean (CI)	Difference between groups Mean (CI)	*p*-value mean difference
Baseline	-	-	-	-	3.3 (2.7 to 3.9)	3.8 (3.2 to 4.5)	0.5 (−1.4 to 0.4)	0.26
3–4 weeks	32 (26)	17 (21)	15 (6 to 24)	<0.05	1.7 (1.1 to 2.4)	1.6 (1.0 to 2.3)	0.1 (−0.8 to 1.0)	0.80
6–7 weeks	58 (28)	32 (29)	26 (15 to 37)	<0.05				
13 weeks	66 (28)	47 (33)	19 (7 to 31)	<0.05	2.6 (2.0 to 3.2)	1.8 (1.1 to 2.5)	0.8 (−0.1 to 1.8)	0.077
26 weeks (6 months)	63 (31)	54 (33)	9 (-3 to 22)	>0.05	2.2 (1.6 to 2.8)	1.7 (1.1 to 2.4)	0.5 (−0.4 to 1.4)	0.29
52 weeks (1 year)	70 (24)	59 (30)	11 (1 to 23)	<0.05	2.5 (1.9 to 3.1)	2.1 (1.5 to 2.8)	0.4 (−0.5 to 1.2)	0.42

*Source*: Authors’ own work

Grey Block van der Windt *et al*. (1998): did not provide baseline measures as means and standard deviations (SD).

Grey Block Rhon *et al*. (2014): did not measure pain outcomes at 6–7 weeks.

CSI, corticosteroid injections; SD, standard deviation; CI, confidence interval.

**TABLE 4:** Results (median and interquartile range) for the measurement of pain in Hay *et al*. (2003).

Variable	Physiotherapy median (IQR)	CSI median (IQR)	*p*-value difference between groups
**Pain during the day**			
Baseline	50 (40–70)	50 (40–60)	>0.05
6–7 weeks	30 (10–40)	30 (10–50)	>0.05
26 weeks/6 months	10 (0–30)	20 (0–30)	>0.05
**Pain during the evening**			
Baseline	50 (30–70)	50 (30–70)	>0.05
6–7 weeks	20 (10–40)	30 (0–60)	>0.05
26 weeks/6 months	10 (0–30)	20 (0–40)	>0.05

Source: Authors’ own work

IQR, interquartile range; CSI, corticosteroid injections.

**TABLE 5:** Results (mean and standard deviation) for the measurement of range of motion in van der Windt *et al*. (1998).

Variable	CSI mean (SD) improvement	Physiotherapy mean (SD) improvement	Mean (95% CI) difference between groups	*p*
**External rotation**				
3–4 weeks	6 (14)	−3 (12)	9 (3 to 14)
6–7 weeks	13 (16)	−2 (14)	15 (9 to 20)	0.002
26 weeks	16 (18)	7 (21)	9 (1 to 16)	
**Abduction**				
3–4 weeks	2 (12)	−3 (13)	5 (0 to 9)
6–7 weeks	4 (11)	−1 (14)	5 (0 to 10)	0.065
26 weeks	9 (12)	7 (17)	2 (−3 to 8)	

*Source*: Authors’ own work

A minus (−) indicates decrease in ROM. SD, standard deviation; CSI, corticosteroid injections; CI, confidence interval.

**TABLE 6:** Results for the measurement of range of motion (%) in Hay *et al*. (2003).

Variable	CSI	Physiotherapy
**Restricted active abduction (% yes)**
Baseline	73%	76%
6–7 weeks	54%	40%
26 weeks/6 months	39%	31%
**Restricted active external rotation (% yes)**
Baseline	9%	21%
6–7 weeks	12%	8%
26 weeks/6 months	8%	7%
**Restricted passive external rotation (% yes)**
Baseline	7%	14%
6–7 weeks	7%	7%
26 weeks/6 months	6%	5%

Source: Authors’ own work

CSI, corticosteroid injections.

**TABLE 7:** Results (mean and 95% confidence interval) for the measurement of shoulder function in Rhon *et al*. (2014).

GRC score (−7 to +7)	CSI mean (95% CI)	Physiotherapy mean (95% CI)	Mean difference (95% CI)	*p*-value of mean difference
1 month	3 (2 to 5)	3 (2 to 5)	0 (−2 to 2)	0.99
3 months	3 (2 to 4)	4 (3 to 5)	1 (−2 to 1)	0.32
6 months	3 (2 to 4)	3 (1 to 4)	0 (−1 to 2)	0.32
1 year	3 (2 to 4)	3 (2 to 4)	0 (−2 to 1)	0.53

*Source*: Authors’ own work

CI, confidence interval; CSI, Corticosteroid injections; GRC, global rating of change.

## Pain

van der Windt *et al*. (1998) assessed pain using the Visual Analogue Scale (VAS) (0–100 mm) and the mean change from baseline at 3- to 4-, 6- to 7-, 13-, 26- and 52-week intervals was recorded and tabulated. A statistically significant difference in favour of the CSI group was recorded at 3–4, 6–7, 13 and 52 weeks (Table 3). Rhon *et al*. (2014) assessed pain using the Numeric Pain Rating scale and found no statistical difference between CSI and physiotherapy groups at the different time intervals (see Table 3).

Hay *et al*. (2003) made use of the VAS (0–100 mm) to measure the level of pain during the day and night at 6 weeks and 6 months (Table 4). The data show that while both the CSI and physiotherapy had a positive effect in decreasing pain, neither had a statistically greater effect (*p* > 0.05).

## Shoulder range of motion

Table 5 shows the mean (SD) improvement in shoulder external rotation and abduction as reported in van der Windt *et al*. (1998). The effect of CSIs on the range of external shoulder rotation was significantly greater than that of physiotherapy at 3–4, 6–7 and 26 weeks (*p* = 0.002). There were no significant differences between the groups for shoulder abduction at the different time points of measurement (*p* = 0.065).

Hay *et al*. (2003) measured active abduction and active and passive external shoulder ROM and recorded the percentage of patients not reaching 180 degrees of shoulder abduction and if they had a restriction of >50% of external rotation compared with the non-involved arm (Table 6). Both physiotherapy and CSIs caused an increase in abduction and external rotation but neither had a greater effect (*p* > 0.05). Physiotherapy had a greater initial effect when measured in week 6, but at 6 months the scores for both groups were comparable. With regards to the passive external rotation, physiotherapy appeared to have a greater effect by 6 months reducing the score by 9% compared with baseline, whereas CSI only reduced the score by 1%.

## Shoulder function

Rhon *et al*. (2014) assessed the improvement of function using the global rating of change (GRC) scale. The results showed that there was a clinically important improvement in functional ability for both the physiotherapy and CSI groups (≥3 points from baseline) at 1-month, 3-month, 6-month and 1-year intervals (Table 7). There were no significant differences between the groups at any of the assessment intervals.

**FIGURE 2:**
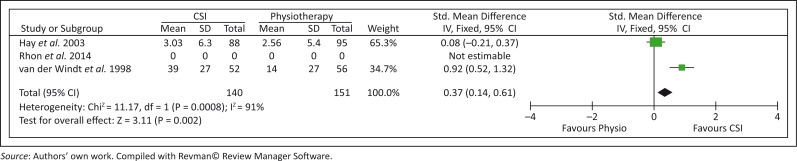
Results for the combined effect for shoulder function at 6–7 weeks.

**FIGURE 3:**
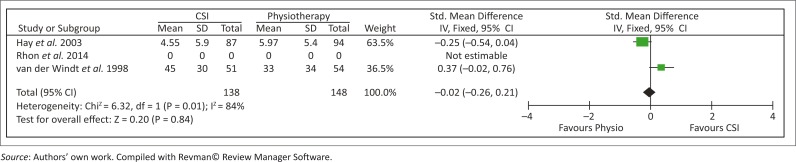
Results for the combined effect for shoulder function at 26 weeks (6 months).

Hay *et al*. (2003) and van der Windt *et al*. (1998) made use of the shoulder disability questionnaire (SDQ) to measure shoulder function. The following forest plots (Figures 2 and 3) show the combined effect of physiotherapy versus CSIs in improving shoulder function measured at 6–7 weeks and 26 weeks (6 months). The overall combined effect at 6–7 weeks (Figure 2) indicated a significant improvement in shoulder function in favour of the CSI group (*p* < 0.0001). However, at 6 months, the overall combined effect indicated no difference between the groups (*p* = 0.84) (Figure 2). At the 52-week (1 year) assessment, van der Windt *et al*. (1998) found no significant differences between the groups [Mean (95% CI) difference between groups: 4 (−10 to 17)].

## Discussion

This is the first systematic review on the effectiveness of physiotherapy compared with CSIs in patients with SIS. Our findings suggested that physiotherapy and CSIs both showed improvement in pain, shoulder ROM and shoulder function in the short term (1–3 months), mid-term (6 months) and long term (12 months). Rhon *et al*. (2014) showed recovery in both groups from baseline up until and including 1 year for pain and function; however, no significant differences were found between the two groups at any time point. Hay *et al*. (2003) showed no difference between the physiotherapy group and the CSIs group with regard to pain, function and ROM as measured at 6–7 weeks and 6 months. A meta-analysis of the combined short-term effect of shoulder function at 6–7 weeks (Hay *et al*. 2003; van der Windt *et al*. 1998) indicated a significant improvement (*p* < 0.0001) in favour of CSI, but by the mid-term follow-up (6 months), the effect was no longer significant (*p* = 0.84). Contradictory results were found in the two articles that measured shoulder ROM (Hay *et al*. 2003; van der Windt *et al*. 1998). From these results, we can conclude that apart from a significant improvement in shoulder function in favour of CSI at the short-term follow-up (6–7 weeks), no evidence was found for the superiority of CSIs compared with physiotherapy in the short term, mid-term and long term.

The article by van der Windt *et al*. (1998) was the only one that showed a significant decrease in day and night pain in the short term and long term; shoulder external ROM in the short term and mid-term as well as shoulder function in the short term and mid-term in favour of the CSI group. Van der Windt *et al*. (1998) came to the conclusion that the short-term differences found between the CSIs and physiotherapy groups mainly resulted from the fast relief of symptoms participants in the CSIs group experienced owing to the anti-inflammatory and analgesic properties of CSIs. Celik *et al*. (2009) compared CSIs and physiotherapy with physiotherapy alone in the treatment of SIS and the purpose of the study was to determine whether or not CSIs played a beneficial role in the management of SIS. At the initial 24-hour follow-up, the CSIs group had a greater reduction in pain scores. At the 3- and 6- week re-evaluation, both groups showed a significant improvement in pain, function, ROM and strength compared with their pre-treatment scores and between-group analyses indicated no significant difference. A RCT conducted by Crawshaw *et al*. (2010) compared CSI and exercise with exercise therapy alone in patients with moderate to severe shoulder pain. They found significantly earlier improvements in pain and functional disability at 1 and 6 weeks in the group given CSI combined with exercise therapy but found no significant difference in the score on the shoulder pain and disability index at 3 months. Crawshaw *et al*. (2010) suggested that if early pain relief is a priority for the patient and physiotherapist, adding local CSI to a course of physiotherapy would seem to be the best management option. The initially significant short-term effect of shoulder function at 6–7 weeks (see Table 8) in favour of CSI in the current study was most likely owing to the anti-inflammatory and analgesic properties of CSIs.

A RCT by Dickens, Williams & Bhamra (2005) included two groups with SIS, one receiving physiotherapy and another continuing with daily activities; however, both groups had received a minimum of three CSIs (80 mg of methylprednisolone acetate) prior to the commencement of the trial. At the 6-month follow-up, the results showed that physiotherapy significantly decreased shoulder pain and increased shoulder strength and function over and above the use of CSIs. CSIs and physiotherapy seem to work in synergy, the former decreases inflammation and pain, while physiotherapy addresses the mechanical problems that may have caused SIS. Dickens *et al*. (2005) found that the combination of CSIs and physiotherapy could be significantly superior to CSIs alone, in improving SIS symptoms to such an extent that the patients in their study no longer require surgery. The potential benefit and detrimental effects of CSI should however be considered before CSIs are administered to patients with SIS. Maman *et al*. (2015) cautioned that the biological basis of the effect of CSIs is still not understood and that optimal dosages, delivery techniques and intervals between injections and post-injection care remain unknown. Their animal study found that a triple methylprednisolone acetate injection significantly weakened the rotator cuff muscles and had a detrimental effect on bone quality in rats (Maman *et al*. 2015). Despite the popularity of CSIs, there is a serious lack of scientific research on the short- and long-term side-effects of CSIs in humans with SIS, and improvement after a CSI may not necessarily be contributed to a decreased progression of SIS. This was demonstrated by Ramírez *et al*. (2014) who found a 17% increase of full-thickness rotator cuff tears 12 weeks after a single CSI (triamcinolone acetate 40 mg), even though patients reported significant improvement in pain symptoms (*p* = 0.0001; VAS score) and shoulder ROM (*p* = 0.002 for forward elevation and external rotation). After an extensive literature search, no evidence on the mid- and long-term effect of CSIs on disease progression in patients with SIS could be found. The use of CSIs to ease initial pain and improve function in patients with SIS should thus be carefully considered.

The strengths of this review are that a systematic search strategy was conducted utilising seven scientific databases. Each step of the review was completed independently by at least two investigators and cross-checked by seven of the eight investigators. In addition, an effort was made to contact the authors of the included articles to acquire point measures and measures of variability, which enabled us to conduct a more thorough analysis and combine and pool data for shoulder function. The three RCTs that qualified for this systematic review were classified as evidence Level II according to the NHMRC and were all of high methodological quality, scoring an average of 7.3/10 on the PEDro scale. Blinding of the therapists and participants in the included articles was not possible owing to the nature of the interventions used. A limitation of the study by van der Windt *et al*. (1998) was that they did not attempt to blind the assessors, which could have led to bias during the evaluation of the different outcomes.

In keeping with the findings of this review, no strong evidence supports the recommendation in favour of CSIs over physiotherapy for the treatment of loss of function, decreased ROM and pain in SIS. The recommended choice of intervention for patients with SIS should therefore take into account the patient (e.g. aversion to injections) and physiotherapist’s preferences as well as the availability of the treatment options (e.g. medical doctors trained in giving CSIs for SIS). It is important to involve the patient in the decision-making process and clearly establish the expectations and preferences of the patient. Since sufficient evidence-based information regarding long-term effects of CSI on the progression SIS is seriously lacking, the use of CSIs to ease initial pain and improve function should be carefully considered and physiotherapy management of SIS thus serve as an effective low-risk option.

## Conclusion

In summary, there is Level II evidence suggesting that besides a significant improvement in shoulder function in favour of CSIs at 6–7 weeks’ follow-up, no evidence was found for the superiority of CSIs compared with physiotherapy for pain and ROM in the short term. The medium- and long-term outcomes for pain, ROM and shoulder function do not favour the use of CSIs over physiotherapy. The management for patients with SIS should therefore take into account the patient and physiotherapist’s preferences as well as the possible long-term beneficial and adverse effects of CSIs on the progression of SIS.
